# ISL1-overexpressing BMSCs attenuate renal ischemia-reperfusion injury by suppressing apoptosis and oxidative stress through the paracrine action

**DOI:** 10.1007/s00018-024-05354-5

**Published:** 2024-07-27

**Authors:** Jiale Wang, Jingwen Wang, Cuinan Lu, Ying Wang, Huanjing Bi, Jin Zheng, Xiaoming Ding

**Affiliations:** Department of Renal Transplantation, Hospital of Nephrology, The First Affiliated Hospital of Xi’an Jiaotong University, 277 Yanta Western Rd, Xi’an, Shaanxi 710061 China

## Abstract

Ischemia-reperfusion injury (IRI) is a major event in renal transplantation, leading to adverse outcomes. Bone marrow mesenchymal stem cells (BMSCs) are novel promising therapeutics for repairing kidney injuries. The therapeutic efficacy of BMSCs with ISL1 overexpression in renal IRI and its underlying mechanism need to be investigated. The unilateral renal IRI rat model was established to mimic clinical acute kidney injury. Rats were injected with PBS, BMSCs-Scrambled or BMSCs-ISL1 via the tail vein at the timepoint of reperfusion, and then sacrificed after 24 h of reperfusion. The administration of BMSCs-ISL1 significantly improved renal function, inhibited tubular cells apoptosis, inflammation, oxidative stress in rats. In vitro, HKC cells subjected to H_2_O_2_ stimulation were pretreated with the conditioned medium (CM) of BMSCs-Scrambled or BMSCs-ISL1. The pretreatment of ISL1-CM attenuated apoptosis and oxidative stress induced by H_2_O_2_ in HKC cells. Our proteomic data suggested that haptoglobin (Hp) was one of the secretory proteins in ISL1-CM. Subsequent experiments confirmed that Hp was the important paracrine factor from BMSCs-ISL1 that exerted anti-apoptotic and antioxidant functions. Mechanistically, Hp played a cytoprotective role via the inhibition of ERK signaling pathway, which could be abrogated by Ro 67-7476, the ERK phosphorylation agonist. The results suggested that paracrine action may be the main mechanism for BMSCs-ISL1 to exert protective effects. As an important anti-apoptotic and antioxidant factor in ISL1-CM, Hp may serve as a new therapeutic agent for treating IRI, providing new insights for overcoming the long-term adverse effects of stem cell therapy.

**Supplementary Information:**

The online version contains supplementary material available at 10.1007/s00018-024-05354-5.

## Introduction

Acute kidney injury (AKI) is an encountered syndrome that leading to rapidly decreased kidney function, characterized by increasing incidence, considerable mortality, high costs and lack of effective treatment [1]. The pathogenesis of AKI involves multiple stressors including hypoxia, inflammation and oxidant injury [2]. Besides causing acid-base or electrolyte disturbances, volume overload, and immune dysfunction, AKI is negatively associated with long-term survival [3, 4]. As the most common cause of AKI, ischemia-reperfusion injury (IRI) could lead to renal tubular epithelial cells and endothelial cells injury, activation of immune response, oxidative damage, and interstitial fibrosis [5, 6]. As an inevitable event in the deceased donor transplantation, IRI contributes to delayed graft function (DGF), acute and chronic organ rejection, and graft failure [7, 8]. Effective therapies are in urgent need to limit IRI and improve graft function.

Mesenchymal stem cells (MSCs) are a subset of multipotent cells, capable of differentiating into cells of multiple lineages, such as osteoblasts, adipocytes, myoblasts, and others [9, 10]. MSCs are known for their hypoimmunogenicity, availability from different tissues, and immunomodulatory capabilities [11, 12]. Numerous studies demonstrated that MSCs could ameliorate kidney injury by supporting renal tubular epithelial cell survival, inhibiting apoptosis, suppressing immune response, and stimulation of angiogenesis [13, 14]. The principal mechanism that MSCs exert the therapeutic efficacy is their paracrine action, through the release of various mediators, including pro-survival and immunosuppressive molecules, growth factors, exosomes, cytokines, and various metabolites [14, 15]. The tumorigenesis and immunogenicity are some of the safety and efficacy concerns of MSCs therapies in clinical use [16, 17]. Extracellular vesicles derived from MSCs could be utilized as cell-free therapeutics, which have shown great immunomodulatory and regenerative functions, thus providing new perspectives for MSCs therapy [18, 19]. In addition, conditioned medium (CM) derived from MSCs have displayed therapeutic efficacy against IRI by inhibition of inflammatory and apoptosis [20–23]. Pretreatment with hypoxia, certain chemical agents or gene modification could enhance the paracrine effects of MSCs to attenuate IRI, which may suggest a better approach to improve the therapeutic benefits of MSCs [24, 25].

Insulin gene enhancer binding protein 1 (ISL1), a LIM homeodomain transcription factor, has been reported to play vital roles in cardiogenesis, neuronal development, cancer progression, and the maturation of islet cells [26–28]. Our previous work demonstrated that ISL1-overexpressing bone marrow mesenchymal stem cells (BMSCs) reduce the apoptosis of grafted islets through the paracrine function [29]. It has been reported that ISL1 overexpression in human MSCs promoted cell survival and improved cardiac function in the model of myocardial infarction [30]. In addition, exosomes derived from ISL1-MSCs were proved its cytoprotective and pro-angiogenesis abilities in ischemic heart disease [31]. However, whether ISL1 overexpression in BMSCs could lead to alleviation of renal IRI and the improvement of renal function has not been investigated.

In this study, we demonstrated the therapeutic effects of ISL1-overexpressing BMSCs (BMSCs-ISL1) in attenuating renal injury and protecting renal function in a rat IRI model. Furthermore, we found that the conditioned medium (CM) of ISL1-CM displayed anti-apoptotic and antioxidant properties in vitro. Our proteomic analyses revealed that secretory proteins haptoglobin (Hp) may be beneficial for human kidney proximal tubular cells (HKC) against H_2_O_2_-induced injury. Moreover, our results indicated that Hp exerted its cytoprotective function by inhibiting extracellular-signal relate kinase (ERK) signaling pathway. Therefore, our study provides a new strategy for boosting the benefits of cell therapy based on MSCs and explores potential protective mechanisms of ISL1-CM.

## Materials and methods

### Isolation and culture of BMSCs

BMSCs were isolated by the whole bone marrow adherent method from Sprague-Dawley rats (male, 60–80 g) as previously described [32]. Briefly, the rats were sacrificed by injecting overdose pentobarbital, and then soaked in 75% ethanol alcohol for 15 min. BMSCs were isolated from the femur and tibia, then suspended with 5 ml of the BMSCs complete medium (Cyagen, Guangzhou, China) and seeded in T25 culture flasks (Corning, NY, USA) at 37 °C in 5% CO_2_ and 95% humidity. After 48 h, the culture medium was replaced. The adherent cells were passaged when 80% confluent. BMSCs from passage 3–5 were used in the experiments. The animals were obtained from the experimental animal center of Xi’an Jiaotong University, Xi’an, China. The protocol was approved by the Xi’an Jiaotong University Committee on Animal Care regulations.

### The construction of BMSCs-ISL1 or BMSCs-scrambled

The detailed description of the construction of BMSCs-ISL1 or BMSCs-Scrambled has been reported in our prior study [29]. In brief, Adenovirus expressing ISL1 and an empty adenoviral vector were constructed from Hanbio Technology Ltd (Shanghai, China). BMSCs were transfected with adenoviruses harboring EGFP or ISL1 according to the manufacturer’s instructions. The titers of adenoviruses used in this study were 1.8 × 10^10^ PFU/ml. The culture medium was replaced 24 h after infection. 72 h after the transduction, the cells were selected by 2.5 µg/ml puromycin (Sigma-Aldrich, St. Louis, USA). The green fluorescence could be detected by a fluorescence microscope (Zeiss, Jena, Germany). BMSCs-ISL1 or BMSCs-Scrambled could differentiate into adipocytes and osteoblasts, identified by Oil Red O staining (Cyagen, Guangzhou, China) and Alizarin Red S staining (Cyagen, Guangzhou, China), respectively. BMSCs-ISL1 and BMSCs-Scrambled were identified by staining with allophycocyanin (APC)-conjugated anti-CD29, phycoerythrin (PE)- conjugated anti-CD90, APC-conjugated anti-CD45 and PE-conjugated anti-CD11b/c monoclonal antibodies (eBioscience, CA, USA). Isotype IgG antibodies (eBioscience, CA, USA) were employed as negative controls. Flow cytometry analyses were performed using a FACS Calibur flow cytometer (BD Biosciences, New Jersey, USA). The data were analyzed using FlowJo software (version 10; Treestar, OR, USA).

### Preparation of conditioned medium (CM) of BMSCs-ISL1 or BMSCs-Scrambled

Upon 80% confluence of BMSCs-ISL1 or BMSCs-Scrambled, the culture medium was removed and then the cells were washed with phosphate-buffered saline (PBS; Servicebio) twice before treated with Iscove’s Modified Dulbecco Medium (IMDM; Procell, Wuhan, China) for 24 h at 37 °C in a humidified atmosphere with 5% CO_2_. The conditioned medium (CM) was then collected, centrifuged at 3000 rpm for 10 min at 4 °C, filtered with 0.22‐µm sterile filters (Millipore, MA, USA) and stored at − 80 °C for further use. Both CM of BMSCs-ISL1 (ISL1-CM) and BMSCs-Scrambled (Scrambled-CM) were diluted with the culture medium of HKC cells at ratio of 1:9 before use.

### Renal IRI model and in vivo treatment

Male Sprague-Dawley rats (140–160 g) were obtained from the experimental animal center of Xi’an Jiaotong University, Xi’an, China and raised in a temperature-controlled and pathogen-free environment with a 12 h light/dark cycle. All animal experiments conducted in this study were in compliance with the regulations by the Xi’an Jiaotong University Committee on Animal Care. Rat IRI model was then established. Briefly, rats were food-deprived for 12 h before the surgery and anesthetized with intraperitoneal injection of pentobarbital (50 mg/kg). The right unilateral nephrectomy was then performed. The left renal pedicle was exposed by lumbodorsal incision and then clamped with a nontraumatic vascular clamp for 60 min. In the Sham group, the right unilateral nephrectomy was performed and the left renal pedicle was exposed but without clamped. During the procedure, the body temperature of rats was maintained at 37 °C using a rectal probe and heat pad. The nontraumatic vascular clamp was removed to restore blood flow, and the kidney was inspected to confirm reperfusion. At the timepoint of reperfusion, rats were administered intravenously via the tail vein with PBS (200 µl), BMSCs-Scrambled (2 × 10^5^ cells in 200 µl PBS) or BMSCs-ISL1 (2 × 10^5^ cells in 200 µl PBS). All rats were euthanized after 24 h of reperfusion, and the blood samples and kidneys were collected for further investigation. Serum levels of creatinine (SCr) and blood urea nitrogen (BUN) were determined by commercial kit reagents (Jiancheng Bioengineering Institute, Nanjing, China).

### Cell culture and treatment

Human kidney proximal tubular cells (HKC) were purchased from the Chinese Academy of Medical Sciences cell bank. The cells were cultured in Dulbecco’s Modified Eagle Medium Nutrient Mixture F-12 (Ham) (DMEM/F12 (1:1), Gibco, USA) containing 10% fetal bovine serum (Gibco), penicillin (100 U/mL, Gibco), and streptomycin (0.1 g/mL, Gibco) at 37 °C in a humidified atmosphere with 5% CO_2_. HKC cells were treated with IMDM, Scrambled-CM or ISL1-CM for 24 h and then stimulated with 250 µM H_2_O_2_ for 4 h. In the subsequent experiments, haptoglobin (MCE, Shanghai, China) was used to treat HKC cells. ERK phosphorylation agonist (S)-2-(4-fluorophenyl)-1-(toluene-4-sulfonyl)-pyrrolidine (Ro 67-7476) was purchased from TargetMol (Massachusetts, USA).

### Cell viability assay and cell death analysis

HKC cells (5,000 cells/well) were seeded into 96-well plates and incubated overnight. HKC cells were then treated with CM or Hp at different concentrations for 24 h before being exposed to H_2_O_2_. The viability of cells was determined through Cell Counting Kit‐8 (CCK-8) assay (Beyotime Biotechnology, China) by following the manufacturer’s instruction. Optical density values were measured at 450 nm on a microplate reader (BioTek, CA, USA). Cell viability was calculated as relative values compared to the control cells.

The apoptosis of HKC cells was measured using an Annexin V-APC and 7-AAD kit (Multisciences, Beijing, China) and detected using flow cytometry, according to manufacturer’s instructions. The percentages of dead cells were analyzed and quantified with FlowJo software (version 10; Treestar, OR, USA).

### Histology analysis and immunohistochemical staining

Kidney tissues were fixed with 4% paraformaldehyde solution, followed by paraffin embedding and slicing (4 μm). The sliced sections were deparaffinized and stained with Periodic Acid-Schiff (PAS) staining. The tubular injury was scored as follows: 0, no damage; 1, < 25%; 2, 25 ~ 50%; 3, 50 ~ 75%; 4, > 75%. The tubular injury score was calculated as the average score from 10 random high-power fields (× 20 objective). The tubular injury was evaluated in a blinded manner. For immunohistochemistry staining, renal sections were incubated in 3% H_2_O_2_ for 10 min, blocked with 5% bovine serum albumin for 1 h at 37 °C and then incubated with primary antibodies against CD68 (GB113109, Servicebio, 1:100) overnight at 4 °C. Then the sections were washed and incubated with horseradish peroxidase-conjugated anti-rabbit secondary antibody for 1 h at room temperature. DAB staining (G1212, Servicebio, China) was performed and the nuclei were counterstained using hematoxylin (Servicebio, China). The sections were observed by an optical microscopy (NikonInstruments, Melville, NY) and 5 random fields (× 40 objective) were selected and measured.

### Terminal deoxynucleotidyl transferase-mediated dUTP nick-end labeling (TUNEL) assay

TUNEL staining was performed on the kidney sections to evaluate cell apoptosis, using one-step TUNEL assay kit (Beyotime Biotechnology, Jiangsu, China). The sections were observed and photographed under a fluorescence microscope (Zeiss, Jena, Germany). 5 fields of vision (× 40 objective) were randomly selected to count the number of TUNEL-positive cells.

### Dihydroethidium (DHE) staining of kidney tissues

Cryosections from frozen kidney tissues (4 μm) were stained with DHE solution (5 µM) (S0063, Beyotime Biotechnology, China) for 60 min in the dark at 37 °C, then washed 3 times with PBS. The sections were counterstained with DAPI (Invitrogen) to label the nucleus. 5 fields of vision (× 20 objective) were randomly selected and then the fluorescence intensity of DHE was quantified by ImageJ software (version 1.53; National Institutes of Health).

### Dichlorodihyfrofluorescein diacetate (DCFH-DA) staining of HKC cells

Reactive Oxygen Species Assay Kit (S0033S, Beyotime Biotechnology, China) was used to measure intracellular level ROS. In brief, HKC cells were incubated with fresh culture medium containing 10 µM DCFH-DA for 15 min, and then washed three times with PBS. The ROS levels were observed using a fluorescence microscope (Zeiss, Jena, Germany) and analyzed by ImageJ software (version 1.53; National Institutes of Health).

### Measurement of oxidative stress

Kidney tissues (100 µg) were homogenized in saline into 10% homogenate. Cell lysates were extracted by radioimmunoprecipitation assay buffer. Superoxide dismutase (SOD) activities and malondialdehyde (MDA) levels were measured in kidney tissues and HKC cells to evaluate oxidative stress using commercial reagent kits (A001-1, Jiancheng Bioengineering Institute, Nanjing, China; G4300-96T, Servicebio, China). In addition, the concentrations of lactate dehydrogenase (LDH) in the serum of rats and supernatants of HKC cells were assessed by the LDH assay kit (S03034, Rayto Life Sciences Co., Ltd, Shenzhen, China).

### Enzyme-linked immunosorbent assay (ELISA)

The levels of haptoglobin released in the conditioned medium were measured by the ELISA kits (Elabscience Biotech, Wuhan, China) in accordance with the manufacturer’s instructions.

### Real-time quantitative polymerase chain reaction (RT-qPCR)

Total RNA was extracted from kidney tissues using TRIzol reagent (Thermo Fisher Scientific, USA). Total RNA (1 µg) was reverse-transcribed into cDNA using reverse transcription polymerase (Roche, Mannheim, Germany). RT-qPCR was performed using the ABI 7500 Fast Real-Time PCR System (Thermo Fisher Scientific, USA) with quantitative SYBR Green PCR Master Mix (Qiagen, Hilden, Germany). The relative mRNA levels of target genes were calculated using the 2^−ΔΔCt^ method. The expression of each gene was normalized to that of Gapdh. The primers are listed in Table S1.

### Western blotting

Proteins were extracted from kidney tissues and HKC cells with RIPA lysis buffer (Beyotime Biotechnology, China) containing protease inhibitor and phosphatase inhibitor (Pierce, IL, USA). To detect haptoglobin in the conditioned medium, methanol–chloroform precipitation method was used [33]. Total protein (25 µg) was subjected to 10% SDS-PAGE gels and subsequently transferred onto polyvinylidene difluoride membranes. After blocking with 5% bovine serum albumin (BSA, Gibco, USA) for 1 h at room temperature, membranes were incubated with primary antibodies, including rabbit anti-ISL1 (1:5000, Novus Biologicals, Colorado, USA, #NBP2-14999), mouse anti-Haptoglobin (1:500, Santa Cruz Biotechnology, USA, #sc-376,893), mouse anti-Bcl-2 (1:1000, Cell Signaling Technology, USA, #15071S), rabbit anti-Bax (1:1000, Cell Signaling Technology, USA, #2772S), rabbit anti-ERK (1:1000, Cell Signaling Technology, USA, #4695S), rabbit anti-phospho-ERK (1:1000, Cell Signaling Technology, USA, #4370S), mouse anti-JNK (1:500, Santa Cruz Biotechnology, USA, #sc-137,019), mouse anti- phospho-JNK (1:500, Santa Cruz Biotechnology, USA, #sc-293,136) and mouse anti-GAPDH (1:5000, Proteintech, 60004-1-Ig) overnight at 4 °C. The membranes were washed with PBS containing Tween 20 and incubated with HRP-conjugated secondary antibodies (1: 5000, Abcam, Cambridge, UK) for 1 h at room temperature. Protein bands were visualized using an enhanced chemiluminescence Western blotting detection kit (Tanon, Shanghai, China) and imaged by a chemiluminescence imaging system (Tanon 5200, Shanghai, China). Three independent biological replicates were performed. All protein expression was normalized to GAPDH. The intensity of each band was assessed using the ImageJ software.

### Statistical analysis

The statistical analyses were performed using GraphPad Prism version 9.0 (GraphPad Inc., CA, USA). All data are presented as the means ± standard deviation (SD). Comparisons between groups were tested using Student’s t test or one-way ANOVA with post hoc Tukey’s test where appropriate. A value of *P* < 0.05 was considered to indicate significance.

## Results

### BMSCs-ISL1 alleviates renal IRI in rats

After adenovirus transfection, BMSCs-ISL1 and BMSCs-Scrambled were obtained. BMSCs-ISL1 and BMSCs-Scrambled showed green fluorescence under a fluorescence microscope 72 h after transfection (Fig. S1a-b). Alizarin Red staining and Oil Red O staining demonstrated their capabilities of differentiating into osteoblasts and adipocytes (Fig. S1c-d). Flow cytometry was performed to detect the surface markers. Both groups of cells expressed phenotypic markers CD29 and CD90, while did not express CD45 or CD11b/c (Fig. S1e). The mRNA and protein expression levels of ISL1 were elevated in BMSCs-ISL1 (Fig. S1f-g). Above results indicated that BMSCs-ISL1 and BMSCs-Scrambled were successfully constructed and maintained the characteristics of BMSCs.

**Fig. 1:**
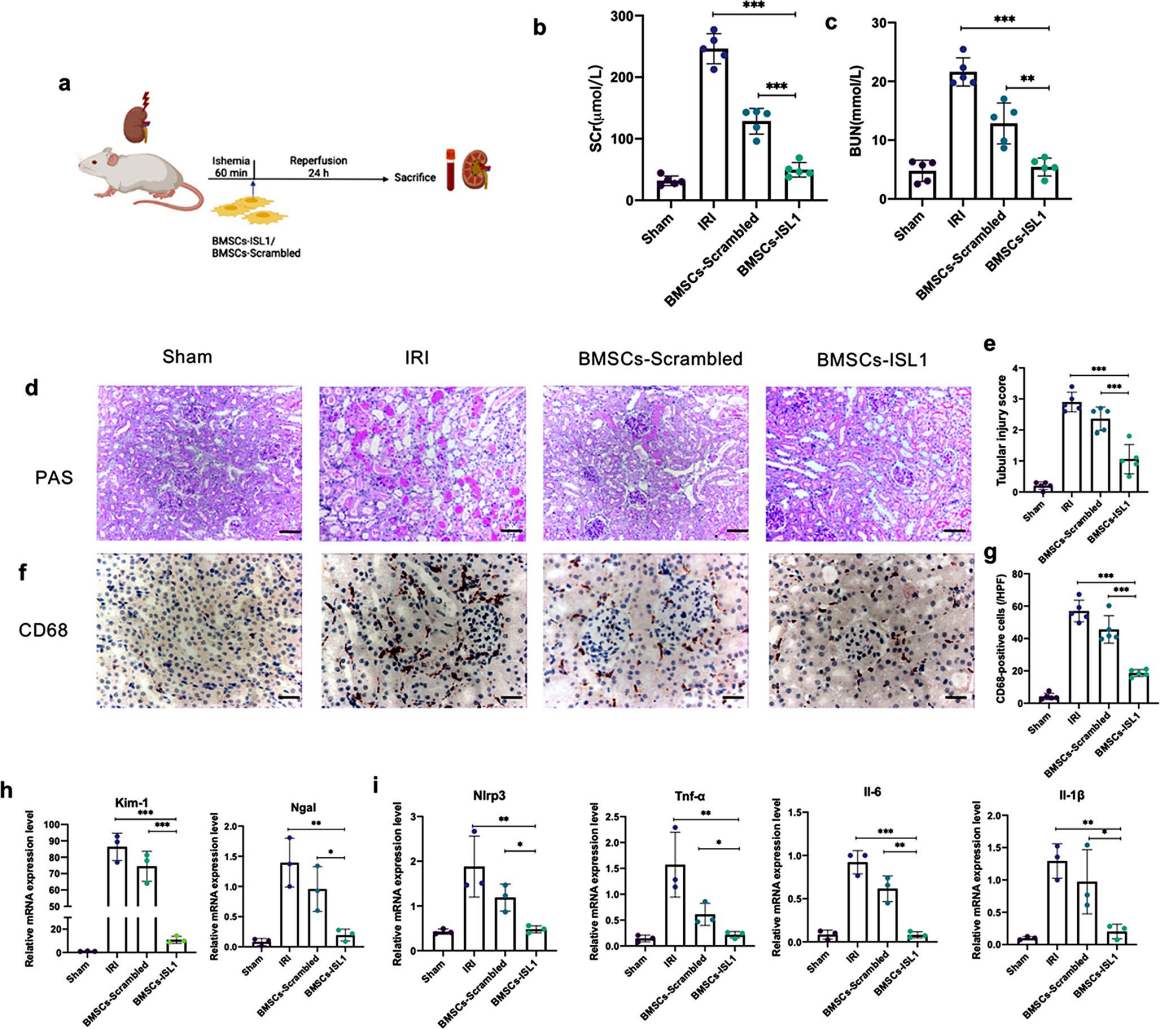
BMSCs-ISL1 attenuate renal dysfunction and tubular injury induced by IRI in rats. **a** Schematic illustration of the animal model in this study. **b-c** The concentrations of SCr and BUN were measured in rats from different groups. The administration of BMSCs-ISL1 improved renal function significantly. (*n* = 5 biological replicates per group). **d** Representative images of PAS staining of the kidneys. Scale bar = 100 μm. **e** Tubular injury scores for histology grading in rats were analyzed. (*n* = 5 biological replicates per group). **f** Representative images of immunohistochemistry staining of CD68. **g** Quantification of CD68-positive cells. Five random fields were chosen from each section. Scale bar = 50 μm. (*n* = 5 biological replicates per group). **h** RT-qPCR analyses of Kim-1 and Ngal mRNA expression levels in renal tissues. (*n* = 3 biological replicates per group). **i** RT-qPCR analyses of renal inflammatory factors (Nlrp3, Tnf-α, Il-6 and Il-1β) mRNA expressions. (*n* = 3 biological replicates per group). ^*^P < 0.05, ^**^P < 0.01, ^***^P < 0.001, ^****^P < 0.0001. Data are presented as mean ± SD. Statistically significant differences were determined by one-way ANOVA with Tukey’s post hoc comparison

To investigate the protective efficacy of BMSCs-ISL1 in vivo, the rats were intravenously injected with PBS, BMSCs-Scrambled or BMSCs-ISL1 at the timepoint of reperfusion. After 24 h, the rats were sacrificed to obtain blood samples and kidney tissues (Fig. 1a). BMSCs-ISL1 treated rats showed improved renal function with markedly lower SCr and BUN levels (Fig. 1b-c). PAS staining showed that IRI caused cast formation, the loss of brush border and tubular necrosis, while BMSCs-ISL1 treatment significantly ameliorated renal damages (Fig. 1d-e). The infiltration of inflammatory cells was assessed by immunohistochemistry staining of CD68. The number of infiltrated macrophages markedly decreased in the BMSCs-ISL1-treated group (Fig. 1f-g). Moreover, the mRNA expression levels of kidney injury markers (Kim-1 and Ngal) and inflammatory factors (Nlrp3, Tnf-α, Il-6 and Il-1β) in the kidney tissues of BMSCs-ISL1 group were lower than those in the kidney tissues of BMSCs-Scrambled group (Fig. 1h-i). Collectively, these results indicated the potential therapeutic effects of BMSCs-ISL1 in protecting renal function and reducing the renal injuries.

### BMSCs-ISL1 reduces apoptosis of renal tubular cells in the renal IRI model

**Fig. 2:**
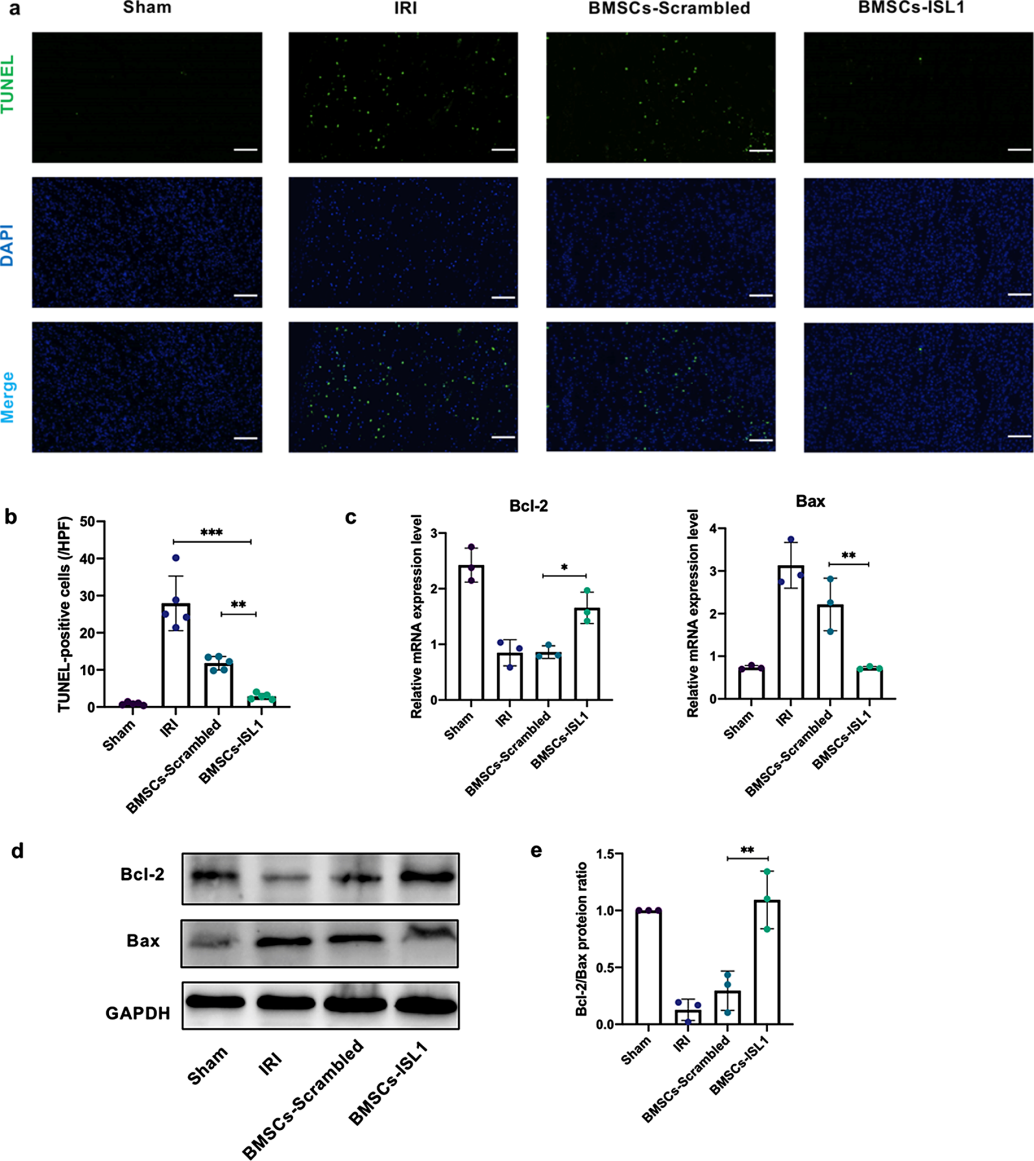
BMSCs-ISL1 reduce IRI injury-induced apoptosis of renal tubular cells. **a** Representative images of TUNEL staining in kidneys of rats. Scale bar = 50 μm. **b** Quantification of TUNEL-positive cells. Five random fields were chosen from each section. (*n* = 5 biological replicates per group). **c** RT-qPCR analyses of Bcl-2 and Bax mRNA expression levels in renal tissues. (*n* = 3 biological replicates per group). **d** Western blot for Bcl-2 and Bax in the kidneys of rats. **e** Quantitative analysis of protein expression levels of Bcl-2 and Bax. (*n* = 3 biological replicates per group). ^*^P < 0.05, ^**^P < 0.01, ^****^P < 0.0001. Data are presented as mean ± SD. Statistically significant differences were determined by one-way ANOVA with Tukey’s post hoc comparison

Apoptosis is one of the mechanisms that contributing to cell death and organ damage in renal IRI [34]. TUNEL staining showed that IRI induced renal tubular cells apoptosis, however, the number of TUNEL-positive cells was lower in the kidneys of BMSCs-ISL1-treated rats (Fig. 2a-b). Besides, apoptotic markers were examined at both mRNA and protein levels. IRI increased Bax mRNA expression level while decreased Bcl-2 mRNA expression level. The administration of BMSCs-ISL1 could reverse the Bax and Bcl-2 mRNA expression levels (Fig. 2c). Similarly, Western blot results indicated that BMSCs-ISL1 upregulated Bcl-2/Bax protein ratio in the kidney tissues (Fig. 2d-e). Taken together, our results showed the anti-apoptotic effects of BMSCs-ISL1 in the rat renal IRI model.

### The conditioned medium (CM) of BMSCs-ISL1 ameliorates H_2_O_2_-stimulated renal tubular cells apoptosis

**Fig. 3:**
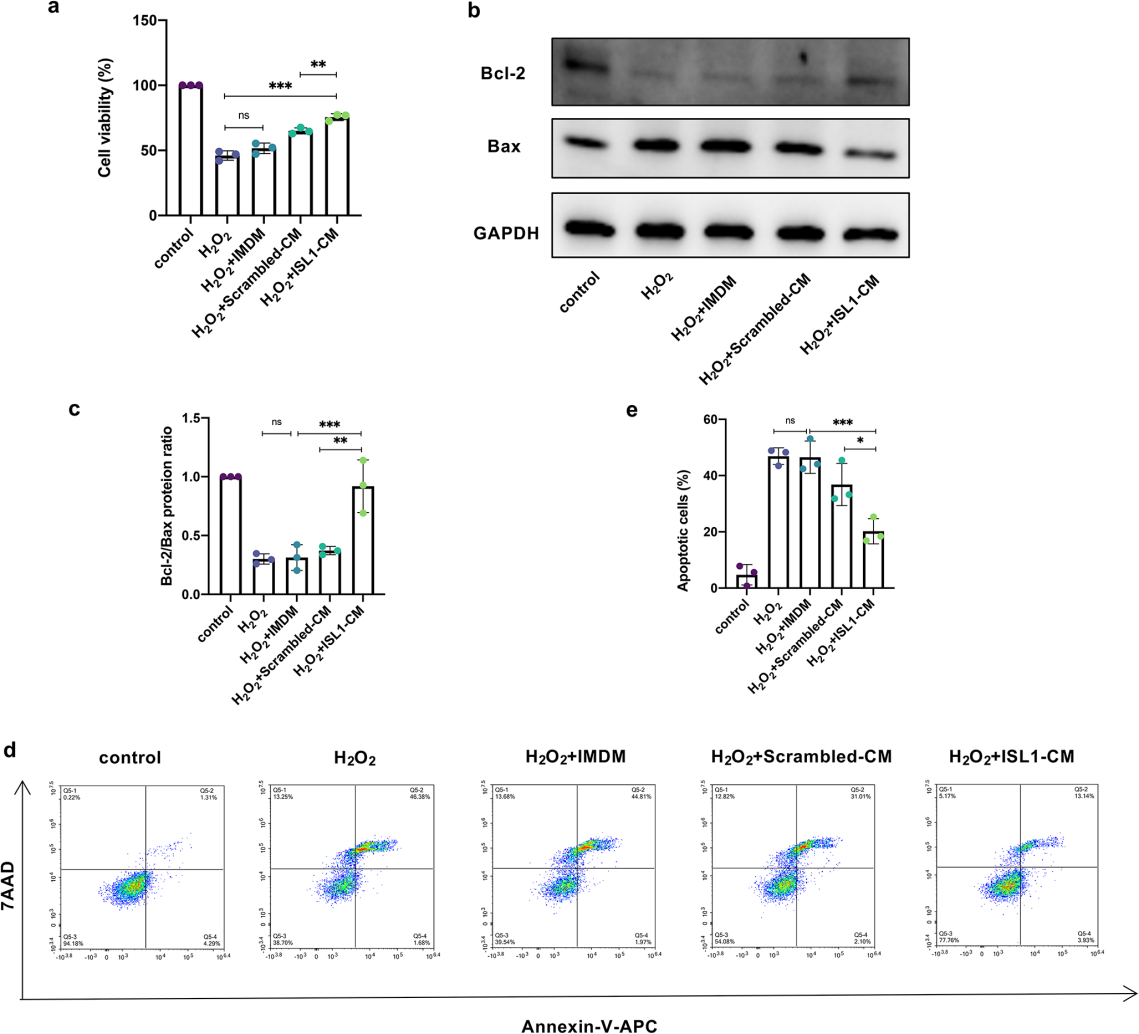
ISL1-CM inhibits cell apoptosis induced by H_2_O_2_. **a** Cell viability of HKC cells was measured by CCK-8 assay. (*n* = 3 biological replicates per group). **b-c** Western blot analyses and quantification of Bcl-2 and Bax protein expression levels in HKC cells. (*n* = 3 biological replicates per group). **d** Flow cytometry with Annexin-V and 7AAD double staining was used to evaluate apoptosis in HKC cells. **e** Statistical analysis of the percentage of Annexin-V-positive cells. (*n* = 3 biological replicates per group). ^*^P < 0.05, ^**^P < 0.01, ^***^P < 0.001, ^****^P < 0.0001; ns, not significant. Data are presented as mean ± SD. Statistically significant differences were determined by one-way ANOVA with Tukey’s post hoc comparison

We collected the CM of BMSCs-Scrambled (Scrambled-CM) and BMSCs-ISL1 (ISL1-CM) to examine whether they could be protective in vitro. HKC cells were pretreated with CM for 24 h before being exposed to H_2_O_2_. Cell viability was determined by CCK-8 assay. We found a significantly elevated cell viability in the ISL1-CM group (Fig. 3a). Western blot results demonstrated that the stimulation of H_2_O_2_ increased the expression of Bax while decreased the expression of Bcl-2 in HKC cells. ISL1-CM significantly increased Bcl-2/Bax ratio compared to the Scrambled-CM (Fig. 3b-c). Subsequently, the results of flow cytometry with Annexin-V-APC/7AAD double staining demonstrated that the ratio of apoptotic cells in ISL1-CM-treated cells decreased dramatically in comparison with the Scrambled-CM-treated cells (20.20% ± 4.49% vs. 36.84% ± 7.51%) (Fig. 3d-e). Thus, our results corroborated that ISL1-CM could protect HKC cells from apoptosis in vitro.

### BMSCs-ISL1 inhibits oxidative stress against IRI in vivo and H_2_O_2_ stimulation in vitro

**Fig. 4:**
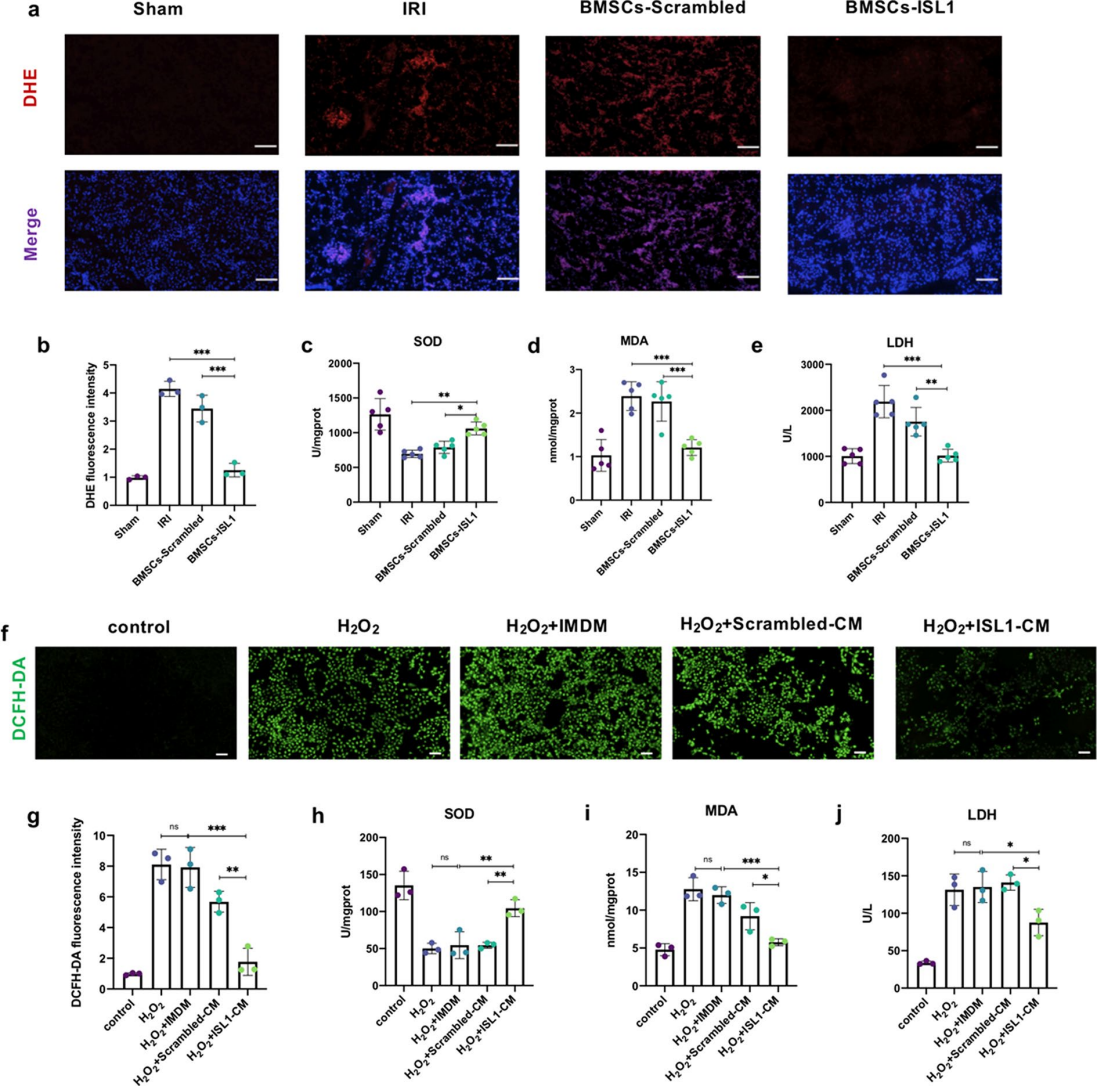
BMSCs-ISL1 decrease oxidative damage in IRI-induced kidneys and H_2_O_2_-stimulated HKC cells. **a** Representative images for DHE staining in the kidneys. Scale bar = 100 μm. **b** Quantification of DHE staining of kidneys. Five random fields were chosen from each section. (*n* = 3 biological replicates per group). **c-d** SOD levels and MDA contents in the kidneys of each group. (*n* = 5 biological replicates per group). **e** Serum LDH levels were measured using the LDH assay kit. (*n* = 5 biological replicates per group). **f** Intracellular ROS levels were measured by DCFH-DA staining. Scale bar = 100 μm. **g** Quantification of the content of ROS in HKC cells. Five random fields were chosen to analyze the fluorescence intensity. (*n* = 3 biological replicates per group). **h-i** The levels of SOD and MDA in HKC cells. (*n* = 3 biological replicates per group). **j** The cellular damage was measured by the LDH release. (*n* = 3 biological replicates per group). ^*^P < 0.05, ^**^P < 0.01, ^***^P < 0.001, ^****^P < 0.0001; ns, not significant. Data are presented as mean ± SD. Statistically significant differences were determined by one-way ANOVA with Tukey’s post hoc comparison

Oxidative stress plays an important role in renal IRI by activating several pathological processes [35]. To investigate whether BMSCs-ISL1 alleviated renal injury by inhibiting oxidative stress. DHE staining of kidney tissues were used to examine the level of reactive oxygen species (ROS) production. The results indicated that the administration of BMSCs-ISL1 led to a significant reduction of ROS (Fig. 4a-b). SOD and MDA levels in kidney tissues were then measured. IRI inhibited SOD activities and increased MDA levels significantly. The treatment of BMSCs-ISL1 was found to increase SOD activities and decrease MDA levels (Fig. 4c-d). Higher serum LDH levels were found after IRI, but BMSCs-ISL1-treated rats had lower serum LDH levels (Fig. 4e). DCFH-DA probe was used to assess the intracellular ROS levels. Fluorescence images showed that H_2_O_2_ increased the ROS production in HKC cells, while ISL1-CM markedly inhibited the ROS production (Fig. 4f-g). Likewise, reduced SOD activities and the accumulation of MDA in HKC cells after H_2_O_2_ stimulation were observed. The treatment of ISL1-CM mitigated oxidative stress by enhancing SOD activities and reducing MDA contents (Fig. 4h-i). Additionally, ISL1-CM significantly inhibited LDH release, suggesting the cell membrane was impaired less severely (Fig. 4j). Collectively, these results suggested that BMSCs-ISL1 protected renal IRI and H_2_O_2_-induced injury owing to its antioxidant properties.

### Haptoglobin is one of the secretory proteins in ISL1-CM that protected HKC cells against H_2_O_2_-induced injury

**Fig. 5:**
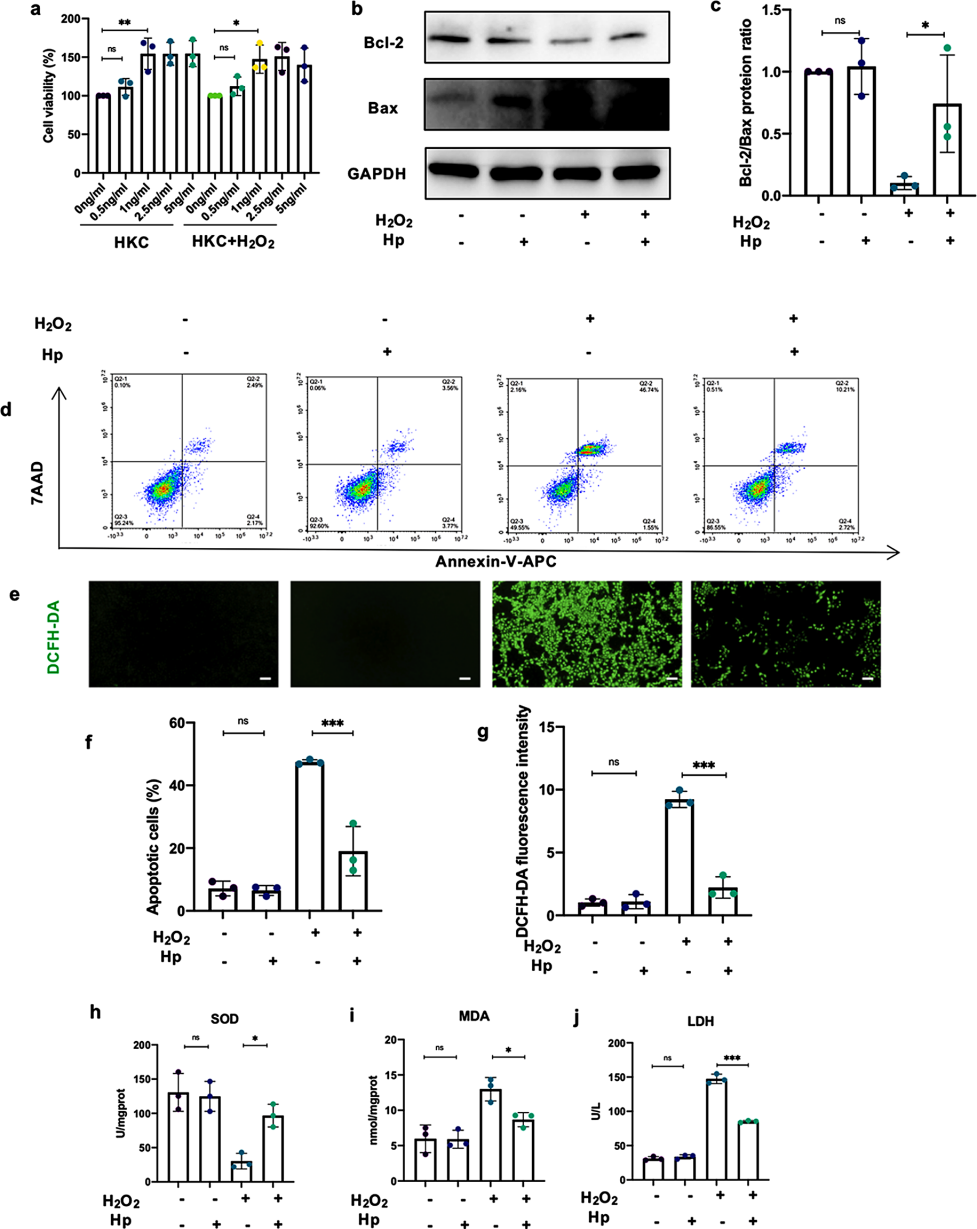
Hp exerts cytoprotective effects against H_2_O_2_-induced injury. **a** Hp treatment elevated cell viability of HKC cells with or without H_2_O_2_ stimulation, assessed by CCK-8 assay. HKC and HKC with H_2_O_2_ stimulation were used as control groups. (*n* = 3 biological replicates per group). **b-c** Western blot showed that Hp increased Bcl-2/Bax protein ratio in HKC cells after H_2_O_2_ stimulation. Hp led to no significant change of Bcl-2/Bax protein ratio without H_2_O_2_ stimulation. (*n* = 3 biological replicates per group). **d**,** f** The percentage of apoptotic cells with or without H_2_O_2_ stimulation and Hp treatment was determined by flow cytometry. (*n* = 3 biological replicates per group). **e** Representative images of HKC cells stained with DCFH-DA. Hp inhibited H_2_O_2_-induced ROS production. Scale bar = 100 μm. **g** Quantification of DCFH-DA. Five random fields were chosen to analyze the fluorescence intensity. (*n* = 3 biological replicates per group). **h-i** The levels of SOD and MDA in H_2_O_2_-stimulated HKC cells after treatment with Hp. (*n* = 3 biological replicates per group). **j** The levels of LDH in supernatants were assessed. (*n* = 3 biological replicates per group). ^*^P < 0.05, ^**^P < 0.01, ^***^P < 0.001, ^****^P < 0.0001; ns, not significant. Data are presented as mean ± SD. Statistically significant differences were determined by one-way ANOVA with Tukey’s post hoc comparison

Our previous study performed proteomics analysis of ISL1-CM and Scrambled-CM and found out 21 secretory proteins that expressed differentially (Table S2) [29]. We focused on haptoglobin (Hp) which showed the highest fold of upregulation in ISL1-CM compared to the Scrambled-CM (Fig. S2a). Enzyme-linked immunosorbent assay (ELISA) showed the markedly increase protein concentrations of Hp in ISL1-CM (Fig. S2b). Western blot showed that the content of Hp was higher in ISL1-CM than Scrambled-CM (Fig. S2c). The mRNA expression level was also upregulated in BMSCs-ISL1 (Fig. S2d). To evaluate the protective effect of Hp, we pretreated HKC cells with different concentrations of Hp for 24 h. The cell viability assessed by CCK-8 method displayed a significant increase at the concentration of 1 ng/ml with or without H_2_O_2_ stimulation. With the concentration increased to 2.5 ng/ml or 5 ng/ml, there was no significant elevation of cell viability compared to the low-dose pretreatment of Hp (Fig. 5a). Western blot results showed that Hp increased Bcl-2/Bax protein ratio when HKC cells were stimulated with H_2_O_2_, while there were no significant changes in the protein expression levels of Bcl-2 and Bax between control group and the control group with Hp pretreatment, suggesting that Hp only exerted protective effect in the presence of H_2_O_2_-induced injury (Fig. 5b-c; Fig. S3c, e). Flow cytometry showed that Hp resulted in the robust reduction in the apoptotic rate of HKC cells exposed to H_2_O_2_ (Fig. 5d, f).

DCFH-DA staining showed Hp significantly reversed the excessive ROS production induced by H_2_O_2_ (Fig. 5e, g). Additionally, Hp increased SOD activities and decreased MDA contents in HKC cells (Fig. 5h-i). LDH release in the culture medium was markedly reduced in the Hp-treated group (Fig. 5j). Collectively, Hp may exert its cytoprotective role by reducing apoptosis and inhibiting oxidative stress.

### Haptoglobin suppresses apoptosis and oxidative stress via ERK signaling pathway

**Fig. 6:**
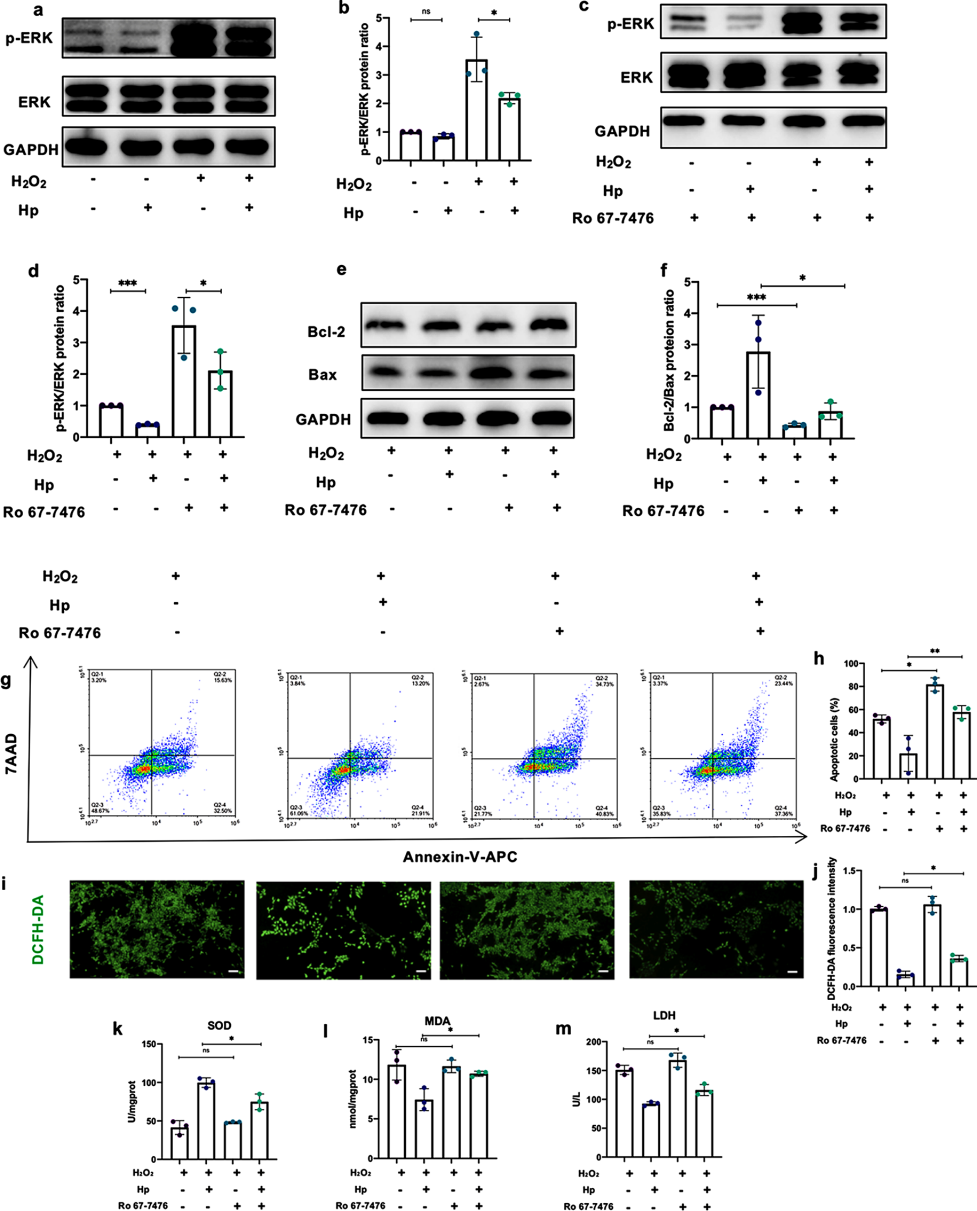
Hp limits H_2_O_2_-induced injury via ERK signaling pathway. **a-b** Western blot and quantitative analysis demonstrated Hp inhibited the phosphorylation of ERK induced by H_2_O_2_ stimulation. (*n* = 3 biological replicates per group). **c-d** Ro 67-7476 (1 µM for 5 min) activated the phosphorylation of ERK, while the phosphorylation of ERK was relieved in the Hp + Ro 67-7476 group compared to the Ro 67-7476 group. (*n* = 3 biological replicates per group). **e-f** Western blot showed that Ro 67-7476 incubation led to lower Bcl-2/Bax protein ratio with the presence of H_2_O_2_. Moreover, Ro 67-7476 + Hp group decreased the Bcl-2/Bax protein ratio compared to the Hp treatment group. (*n* = 3 biological replicates per group). **g-h** Ro 67-7476 + Hp treatment leaded to more apoptotic cells than the Hp treatment group, assessed by flow cytometry. (*n* = 3 biological replicates per group). **i-j** DCFH-DA staining images showed that Ro 67-7476 attenuated the antioxidant effect of Hp. (*n* = 3 biological replicates per group). **k-l** The SOD and MDA levels in HKC cells of different groups were measured. (*n* = 3 biological replicates per group). **m** LDH levels of each group in the cell supernatant. (*n* = 3 biological replicates per group). ^*^P < 0.05, ^**^P < 0.01, ^***^P < 0.001, ^****^P < 0.0001; ns, not significant. Data are presented as mean ± SD. Statistically significant differences were determined by one-way ANOVA with Tukey’s post hoc comparison

ERK signaling pathway participating in apoptosis and ROS stress has been widely investigated [36, 37]. The results of Western blot showed that Hp inhibited the ERK phosphorylation induced by H_2_O_2_ but had no effect on the phosphorylation level of c-Jun N-terminal kinase (JNK) (Fig. 6a-b, Fig. S3a-b). Without the stimulation of H_2_O_2_, Hp treatment did not significantly change the phosphorylation of ERK (Fig. S3c-d). Subsequently, Ro 67-7476, the ERK phosphorylation agonist, was applied to confirm the correlation among Hp, ERK signaling pathway and the cytoprotective functions of Hp. The results of Western blot showed that Ro 67-7476 (1 µM for 5 min) caused the activation of p-ERK (Fig. S3f-g), which was consistent with other previous studies [38, 39]. H_2_O_2_ and Ro 67-7476 treatment led to markedly elevated p-ERK/ERK protein ratio compared with the H_2_O_2_ group, while Hp still inhibited the phosphorylation of ERK (Fig. 6c-d). Without the stimulation of H_2_O_2_, the treatment of Hp displayed not significant inhibition of the phosphorylation of ERK which was activated by Ro 67-7476 (Fig. S3c-d).

Notably, Ro 67-7476 promoted the apoptosis of HKC cells that were exposed to H_2_O_2_, assessed by decreased Bcl-2/Bax protein ratio and flow cytometry (Fig. 6e-h). Moreover, the incubation of Ro 67-7476 partially offset the protective effects of Hp. That is to say, Hp + Ro 67-7476 group displayed more apoptotic cells and a lower Bcl-2/Bax protein ratio compared to the Hp treatment group (Fig. 6e-h). Meanwhile, oxidative stress levels were dramatically attenuated by Hp but reversed by Ro 67-7476. The simultaneous application of Ro 67-7476 and Hp on HKC cells increased the fluorescence intensity of DCFH-DA staining, the levels of MDA in cell lysates, the levels of LDH in cell supernatants, while decreased the levels of SOD in cell lysates, compared to the Hp-treatment group (Fig. 6i-m). Taken together, our results demonstrated that Hp suppressed apoptosis and oxidative stress by inhibiting ERK signaling pathway, while Ro 67-7476 offset its anti-apoptotic and antioxidant effects.

## Discussion

Kidney transplantation is invariably associated with renal damage, including IRI [40]. Renal IRI is the major cause of significant AKI, associated with high mortality and adverse long-term outcomes in kidney transplantation [1, 34]. Despite major progress has been made in the understanding of IRI, effective strategies to prevent allograft damage are still lacking and urgently needed [14, 35]. Intensive studies of MSCs have provided promising therapeutic strategies for the treatment of AKI [41]. However, concerns about the safety and effectiveness of MSCs therapy have been raised. MSCs might embed in the lung and fail to migrate to the injured kidney [42]. Most of MSCs undergo apoptosis after systemic infusion, due to the adverse local microenvironment, which impairs the positive effects of MSCs [43]. The transplanted BMSCs may acquire malignancy and induce tumors in mice models [44]. The administration of MSCs in human subjects with AKI has shown safety but limited efficacy [10]. Hence, further investigations are in great need of to confirm specific therapeutic mechanisms and promote the safe use of MSCs-based cell therapy. Numerous studies demonstrated that MSCs with gene modification, pretreatment, or cultured in hypoxic conditions not only could enhance the survival and migration, paracrine effects, and injury repair efficacy, but also possess minimal adverse impacts and systemic toxicity, which provide the novel strategy to boost MSCs into clinical use [17, 25, 45–48].

ISL1 has been shown the pivotal role in embryogenesis of heart and pancreatic islets [49]. ISL1 expression in endothelial cells promoted their angiogenic properties and IL-1b and VEGF secretion [50]. Our previous work demonstrated that BMSCs overexpressing ISL1 promoted the survival of grafted islets through paracrine function [29]. Besides, ISL1 overexpression MSCs and their derived exosomes have been shown to promote angiogenesis and protect endothelial cells in the myocardial infarction model [30, 31]. In this study, we demonstrated that BMSCs-ISL1 significantly improved renal function, inhibited the mRNA expression levels of inflammatory factors, inhibited apoptosis and oxidative stress induced by IRI. The dose of BMSCs we used in this study (2 × 10^5^ cells) was lower than previously published studies [25, 43, 51], which implied that BMSCs-ISL1 were more beneficial than BMSCs-Scrambled whose therapeutic effects were rather limited. Whether low dose of MSCs could be effective is still controversial, while high dose infusion may cause side effects such as microvascular embolization and tumorigenesis. The appropriate transplantation dose of MSCs is still necessary to further explore [41].

Since most infused MSCs are entrapped in lung and only a small portion of infused cells are able to migrate to the injured sites, the beneficial effects MSCs exerted are primarily due to their paracrine actions [48, 52]. Previous study reported that ISL1-hMSCs expressed elevated levels of monocyte chemoattractant protein-3 (MCP3), thus enhanced the survival and angiogenesis properties of human umbilical vein endothelial cells [53]. Insulin-like growth factor binding protein 3 (IGFBP3) was found higher in the CM of ISL1-hMSCs, which played an important role in the anti-apoptosis efficacy [30]. Our previous work comprehended the impact of ISL1 on the paracrine actions of BMSCs based on proteomic and metabolomic analyses, revealed that BMSCs-ISL1 reduced islet cells apoptosis through exosomes carrying anillin (ANLN), secretory proteins inhibin beta A chain (INHBA), and metabolites caffeine, verified by ChIP-qPCR [29]. In this study, the CM of BMSCs-ISL1 or BMSCs-Scrambled was collected and then used to treat HKC cells. The sudden increase of oxygen could cause rapid injury to the kidney grafts after the restoration of circulation, thus H_2_O_2_, a primary kind of ROS, was used to stimulate HKC cells to simulate the excess ROS production in vivo during IRI [6, 54, 55]. Surprisingly, ISL1-CM was proved to decrease the apoptotic effect and oxidative stress induced by H_2_O_2_ in HKC cells. In brief, previous studies and our work demonstrated that the paracrine actions played a vital role in the beneficial effects of MSCs-ISL1.

Hp is an acute phase plasma protein that captures and binds hemoglobin (Hb) with the highest affinity [56]. The Hp-Hb complex is subsequently cleared from circulation, preventing the oxidative damage in tissues and cells [57, 58]. Besides, Hp is also an angiogenic factor and anti-inflammatory modulator, plays a critical role in various biological processes [59, 60]. Hp is also known as the scavenger of cell-free hemoglobin (CFH), while studies have demonstrated that administration of CFH could lead to severe AKI in renal IRI and septic mice models [61, 62]. The infusion of Hp prevents kidney dysfunction, kidney injury after acute hemolysis as well as blood transfusion [63, 64]. It has been reported that Hp was a cytoprotective protein in kidney, whose mRNA and protein expression levels were applied to assess the protective impact of the agents [65, 66]. Undetectable plasmatic Hp is an independent risk factor for major adverse kidney events (MAKE) and AKI in patients with severe burns, which may be considered as a biomarker for predicting AKI [67]. While high plasma concentrations of Hp might play a protective role from AKI in critically ill patients [68]. In Japan, plasma-derived Hp was approved and clinically used for the treatment of hemolysis due to extracorporeal circulation, burn injuries, trauma, and blood transfusions that could lead to AKI [69, 70]. A retrospective observational study indicated that Hp administration was independently associated with lower risk of AKI in cardiovascular surgery patients, suggesting the renoprotective role of Hp [71]. The low dose administration of Hp (0.5 mg on days 0 and 3) to Shiga-toxin induced mice displayed partial beneficial effects by only reducing the platelet deposition and neutrophil recruitment in kidney, but had no significant influences on the NGAL plasma levels, the PAS staining, and the immunohistochemical analysis of KIM-1 as well as cleaved caspase-3, compared to the mice subjected to Shiga-toxin which were treated with vehicle [72]. Whether Hp may serve as a therapeutic approach in renal IRI has not been investigated, therefore, we will explore the underlying mechanism of Hp in IRI in vivo in the future.

Numerous studies have shown the anti-inflammatory effects and antioxidative properties of Hp, however, the effect of Hp on H_2_O_2_-induced injury in HKC cells still remains unclear [73, 74]. In this study, Hp was found to be one of the secretory proteins that upregulated in ISL1-CM compared to the Scrambled-CM. ELISA and Western blot showed that the release of Hp increased in the ISL1-CM, consistent with proteomic data. The pretreatment of Hp elevated cell viability of HKC cells with or without H_2_O_2_ stimulation. Furthermore, Hp sharply decreased the apoptotic rate and protected HKC cells from oxidative stress that induced by H_2_O_2_. All aforementioned results demonstrated that Hp was an anti-apoptotic and antioxidant paracrine factor from ISL1-BMSCs. ERK has been implicated in cell survival and apoptosis in response to IRI and the H_2_O_2_-induced injury [75, 76]. For instance, taraxasterol protected renal IRI from oxidative stress, inflammation, and apoptosis by suppressing the phosphorylation of ERK and JNK signaling pathways [77]. Dexmedetomidine attenuated hepatic IRI by inhibiting ERK, JNK and p38 phosphorylation [78]. In this study, the decreased phosphorylation level of ERK after Hp treatment was observed, while there is no significant change in the phosphorylation level of JNK. In addition, we conducted ERK agonist (Ro 67-7476) to HKC cells exposed to H_2_O_2_. The results showed that the activation of ERK impaired the protective effects of Hp. Taken these together, our results demonstrated that Hp inhibited apoptosis and oxidative stress via the inhibition of ERK pathway. There may be a complicated crosstalk of the anti-apoptotic and antioxidant mechanisms of Hp in H_2_O_2_-induced injury. The properties and potential molecular signaling mechanisms of Hp in various pathological conditions still need to be further explored.

In conclusion, our findings demonstrated that the administration of BMSCs-ISL1 improved renal function, suppressed apoptosis and oxidative stress in the rat IRI model. However, the tumorigenic risk of MSCs therapy is still a major concern. Long-term studies to verify the safety and effectiveness are still of great necessity. ISL1-CM was proved to attenuate apoptosis and oxidative stress induced by H2O2 in HKC cells. Our proteomics analysis and subsequent in vitro experiments demonstrated that Hp was the important paracrine factor that protected HKC cells, which suggested that Hp might be a novel therapeutic approach that limits apoptosis and oxidative stress in IRI models, thus providing insights to allow cell-free treatments to address lone-term adverse impacts.

### Electronic supplementary material

Below is the link to the electronic supplementary material.

Supplementary Material 1

Supplementary Material 2

Supplementary Material 3

Supplementary Material 4

## Data Availability

All data associated with this study are present in the paper and/or the Supplementary Materials.
