# Supplementary figures and images for: ISL1-overexpressing BMSCs attenuate renal ischemia-reperfusion injury by suppressing apoptosis and oxidative stress through the paracrine action
Source: Cell Mol Life Sci. 2024 Jul 27;81(1):312. doi: 10.1007/s00018-024-05354-5 (PMC11335236; doi:10.1007/s00018-024-05354-5)

Figure S1

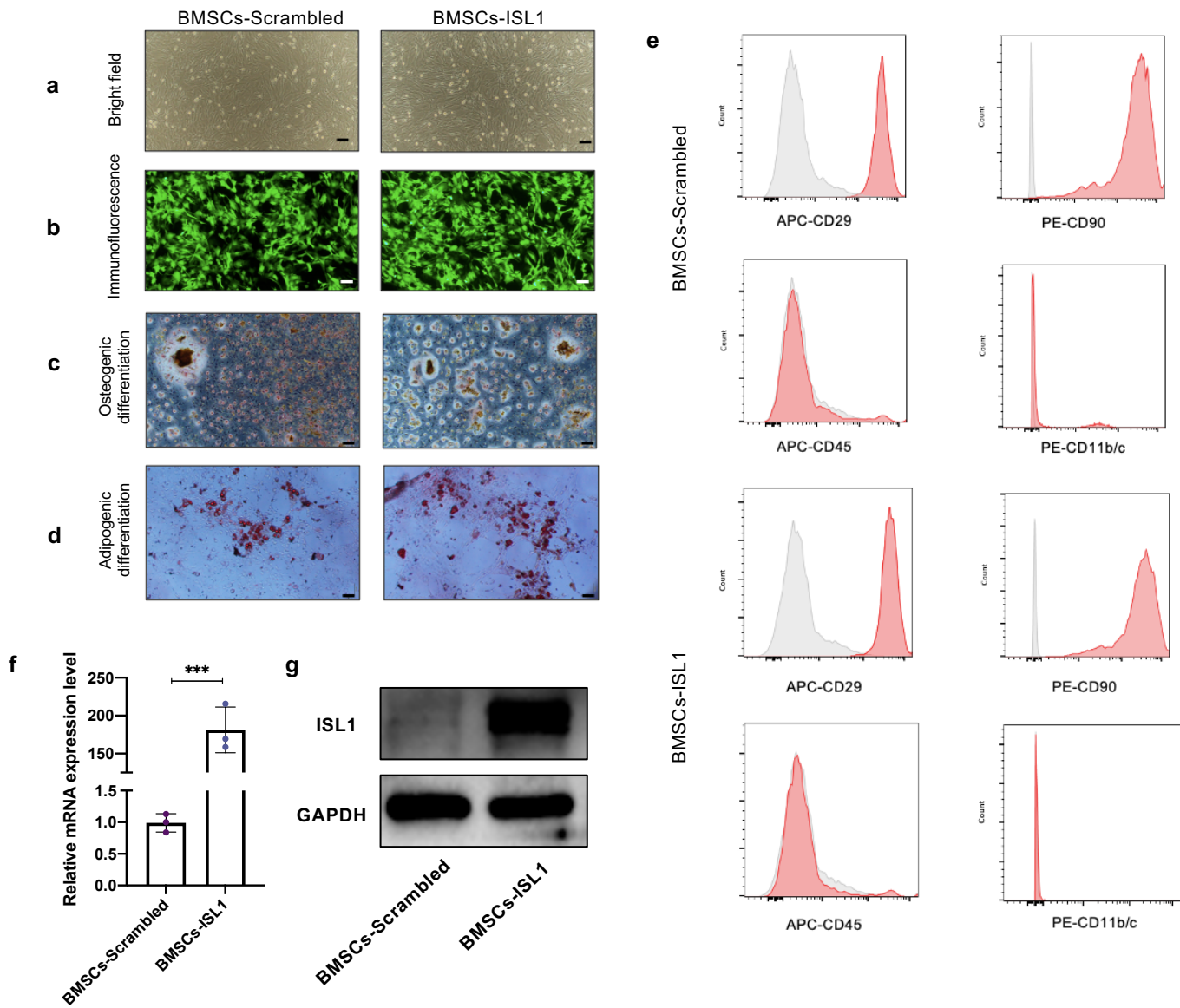

Figure S2

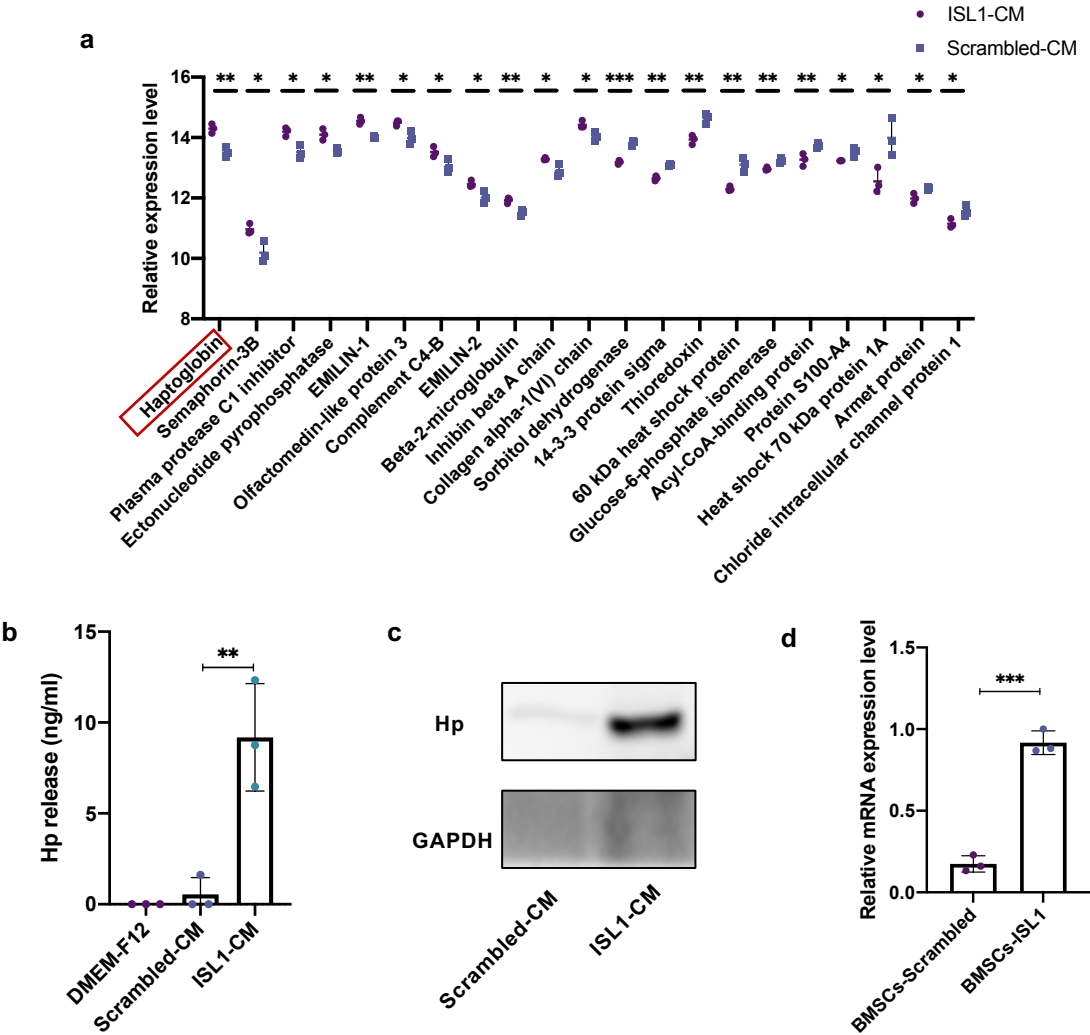

Figure S3

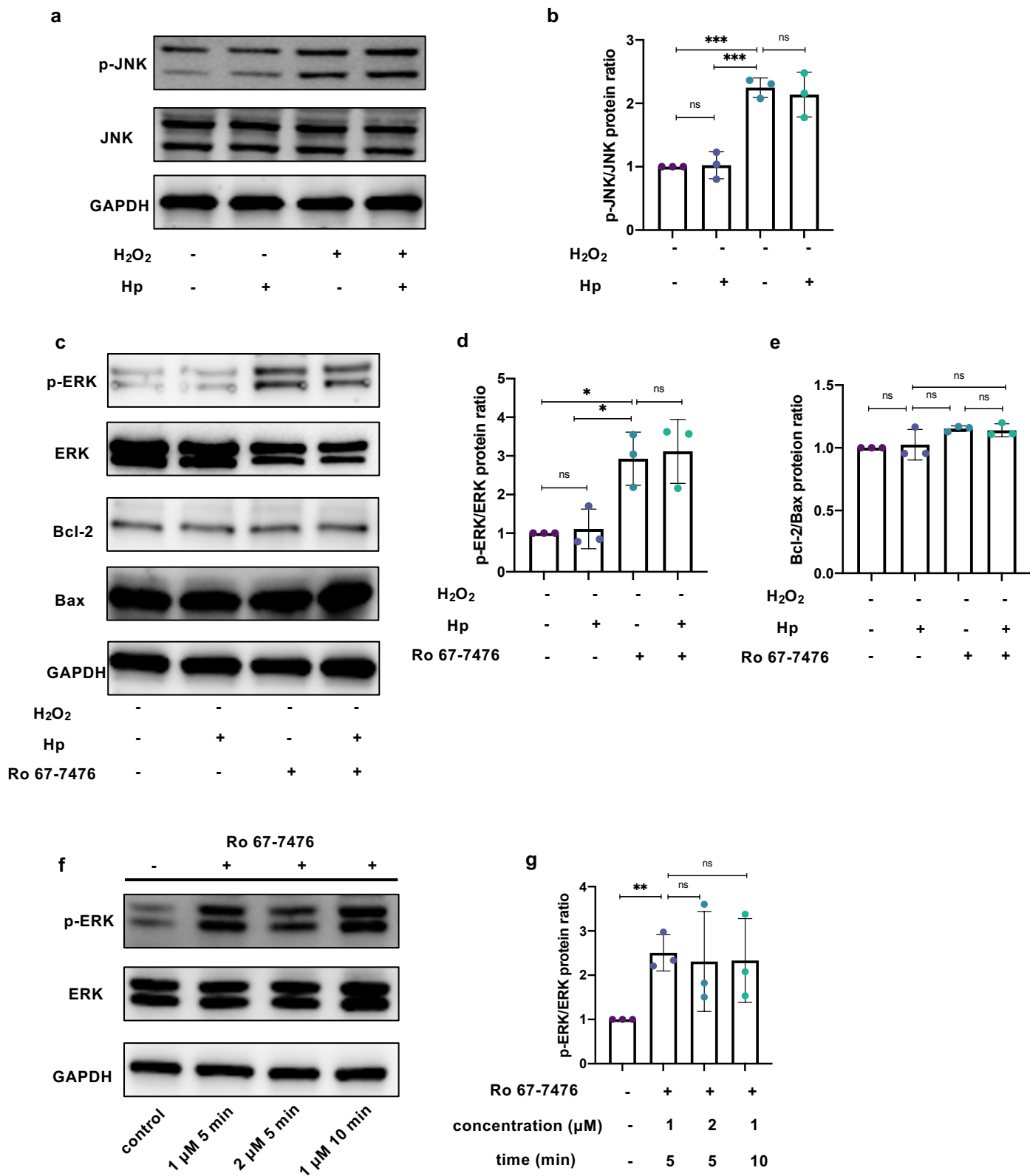

Supplement: Supplementary file 3 — Supplementary Material 3 [file 18_2024_5354_MOESM3_ESM.pdf]
